# Distinct small RNAs are expressed at different stages of *Phytophthora capsici* and play important roles in development and pathogenesis

**DOI:** 10.3389/fgene.2024.1296533

**Published:** 2024-06-11

**Authors:** Shan Zhong, Sicong Zhang, Yang Zheng, Qinghua Zhang, Fangmin Liu, Zhiwen Wang, Xili Liu

**Affiliations:** ^1^ Sanya Institute of China Agricultural University, Sanya, China; ^2^ Department of Plant Pathology, College of Plant Protection, China Agricultural University, Beijing, China

**Keywords:** oomycetes, *Phytophthora capsici*, sequencing, small RNAs, gene silencing, development, infection

## Abstract

Small RNAs (sRNAs) are important non-coding RNA regulators that play key roles in the development and pathogenesis of plant pathogens, as well as in other biological processes. However, whether these abundant and varying sRNAs are involved in *Phytophthora* development or infection remains enigmatic. In this study, sRNA sequencing of 4 asexual stages of *Phytophthora capsici* (*P. capsici*), namely, as mycelia (HY), sporangia (SP), zoospores (ZO), cysts (CY), and pepper infected with *P. capsici* (IN), were performed, followed by sRNA analysis, microRNA (miRNA) identification, and miRNA target prediction. sRNAs were mainly distributed at 25–26 nt in HY, SP, and ZO but distributed at 18–34 nt in CY and IN. 92, 42, 176, 39, and 148 known miRNAs and 15, 19, 54, 13, and 1 novel miRNA were identified in HY, SP, ZO, CY, and IN, respectively. It was found that the expression profiles of known miRNAs vary greatly at different stages and could be divided into 4 categories. Novel miRNAs mostly belong to part I. Gene ontology (GO) analysis of known miRNA-targeting genes showed that they are involved in the catalytic activity pathway, binding function, and other biological processes. Kyoto Encyclopedia of Gene and Genome (KEGG) analysis of novel miRNA-targeting genes showed that they are involved in the lysine degradation pathway. The expression of candidate miRNAs was validated using quantitative reverse transcription–polymerase chain reaction (qRT–PCR), and miRNAs were downregulated in *PcDCL1* or *PcAGO1* mutants. To further explore the function of the detected miRNAs, the precursor of a novel miRNA, miR91, was knockout by CRISPR-Cas9, the mutants displayed decreased mycelial growth, sporangia production, and zoospore production. It was found that 503142 (Inositol polyphosphate 5-phosphatase and related proteins) can be predicted as a target of miR91, and the interaction between miR91 and 503142 was verified using the tobacco transient expression system. Overall, our results indicate that the diverse and differentially expressed sRNAs are involved in the development and pathogenesis of *P. capsici.*

## 1 Introduction

*Phytophthora capsici* (*P. capsici*) is a representative oomycete plant pathogen (Kamoun et al., 2015). The hosts of *P*. *capsici* include about 70 vegetable crops, such as pepper, cucumber, cucurbits, snap, lima beans, and tomato, and it can cause serious losses to agriculture and natural ecosystems (Hwang and Kim, 1995; Lamour et al., 2012; Du et al., 2023; Yang et al., 2023). Asexual lifecycle of *P*. *capsici* dominates in nature, sporangia are produced from mycelia and release zoospores, and these zoospores can transform into cysts that infect plants. The cysts and sporangia are the main resources for infection establishment and spread (Ristaino and Johnston, 1999; Tyler, 2007; Granke et al., 2012). Therefore, the illumination of the development mechanism of *Phytophthora* is urgent.

RNA interference (RNAi) is a common phenomenon in eukaryotes and is regulated by small RNAs (sRNAs). sRNAs of 20–40 nt can regulate gene expression at the transcriptional and post-transcriptional levels (Olivier and Judith, 2004; Zhou et al., 2012; Dou et al., 2013). sRNAs are generated by class III ribonuclease (RNase) Dicer or Dicer-like proteins and can be divided into microRNAs (miRNAs) and small interfering RNAs (siRNAs) according to their origination and formation mechanisms. They can also both be designated as functional sRNAs when loaded into RNA-induced silencing complex (RISC) and can subsequently induce RNAi (Jin and Zhu, 2010; Czech and Hannon, 2011; Claycomb, 2014). miRNAs are usually endogenous RNAs of 20–26 nt, and they have been identified in both metazoans and plants, in which they play diverse regulatory roles in developmental timing, organ development, stress response maintenance, and cell differentiation (Kloosterman and Plasterk, 2006; Zhang et al., 2006; Stefani and Slack, 2008; Liebsch and Palatnik, 2020). They are the most conserved and important sRNAs in endogenous gene expression regulation. Recent studies mainly focused on the function of miRNAs in the infection process of microbes; such miRNAs were found in *Puccinia striiformis* f. sp. *tritici*, *Verticillium dahliae*, *Valsa mali*, and *Fusarium oxysporum* and could repress the expression of fungal virulence genes or plant resistance genes (Jin et al., 2019; Ji et al., 2021; Xu et al., 2022). Moreover, it is shown that miRNAs are indispensable regulators of the plant response to stresses (Zhang et al., 2020).

A few microRNAs (miRNAs) have been identified in *Phytophthora infestans*, and the expression of these miRNAs is elevated dramatically during infection (Cui et al., 2014). In addition, only one miRNA (miR8788) was confirmed in *Phytophthora*. Other sRNAs have been found in the Argonaute1 (AGO1) immunoprecipitation fraction, they are 20–22 nt and have a 5′ C preference. Moreover, AGO4 mainly binds 24–26 nt sRNAs and has a 5′ U preference. This indicates that sRNAs exist in *Phytophthora* species, and different AGOs have different loading preferences (Åsman et al., 2016). In *Phytophthora parasitica*, there are 2 distinct types of sRNAs clustered into 25–26 nt sRNAs and 21 nt sRNAs. Among them, the 21 nt sRNAs do not mediate gene silencing, while the 25–26 nt sRNAs are associated with the efficient silencing of their targets (Jia et al., 2017). However, whether these abundant and markedly different sRNAs are involved in *Phytophthora* development or infection remains enigmatic.

Development and cell differentiation depend on the precise regulation of genes, and miRNAs are known to play important roles in this process in mammals, plants, and nematodes (Navarro et al., 2006; Chen et al., 2011; Bruscella et al., 2017; Chandra et al., 2017; Uri et al., 2023). However, few studies have focused on sRNAs’ function in the development and pathogenesis of plant pathogens, especially of the *Phytophthora* genus. The objectives of this study were thus to 1) investigate the characterization of endogenous sRNAs from different stages of *P*. *capsici*, 2) explore the biological function of known and novel miRNAs, and 3) determine the functions of a novel miRNA, miR91, and verify the interaction between miR91and its target. This study can provide a theoretical basis for the control of oomycete disease based on RNAi.

## 2 Materials and methods

### 2.1 Sampling for the sRNA sequencing

The *P*. *capsici* strain LT1534 (provided by Kurt Lammour, Tennessee State University, Nashville, OR, United States) was routinely cultured on a 5% (v/v) cleared carrot juice agar (CA) medium with 0.01% (w/v) CaCO_3_ in a 90-mm petri dish at 25°C in the dark. For the HY sample, 1-day-old, 2-day-old, and 3-day-old mycelia grown on cellophane on the CA plate were collected and pooled. Sporangia were washed from the plates cultured under light for 5 days and filtrated with a Miracloth (EMD Millipore Corp., 2913897: Billerica, MA, United States). The supernatant was discarded after centrifugation at 1,500 g for 5 min and resuspended with 1 mL of TRIzol. After another round of centrifugation, the precipitate was the SP stage sample (containing a small amount of mycelium). Sterile water was added to the plates that produced sporangia at 4°C for 0.5 h and at room temperature for 0.5 h to collect the ZO suspension. The zoospores were vortexed for 1 min to obtain cysts. Pepper (Xicheng Ox Horn) was grown in a greenhouse at 25°C, 80% relative humidity, and a 16 h light and 8 h dark photoperiod. For the IN sample, the roots of 6 week old pepper seedlings were inoculated with 10 mL of a zoospore suspension, and 3 cm rhizome tissues of the diseased seedlings were collected and cut into pieces after 12 days of inoculation (for *P. capsici* sRNAs enrichment). Healthy areas nearly lesion are also included in the sampling (which could include the sRNAs expressed in early infection stage).

### 2.2 RNA isolation and small RNA library construction

Total RNAs were extracted from the above 5 samples using the TRIzol reagent according to the manufacturer’s protocol (Invitrogen, United States). The total RNAs were sent to the Beijing Genomics Institute (BGI) for small RNA library construction and next-generation sequencing. The quality and quantity of the total RNAs were measured using the Agilent2100 Bioanalyzer and ABI StepOnePlus Real-Time PCR System.

### 2.3 Analysis of known and novel microRNAs

Sequencing was performed using Illumina HiSeq4000 according to the BGI standard process. The genome and gene annotation data of *P*. *capsici* were referenced against the NCBI database (ftp://ftp.ncbi.nlm.nih.gov/genomes/) and *P*. *capsici* genome database (https://genome.jgi.doe.gov/pages/search-for-genes.jsf?organism=Phyca11). RNAs (rRNAs, tRNAs, piRNAs, degradation fragments of exons and introns) were removed after searching with BLAST against the GenBank (http://www.ncbi.nih.gov/Genbank/) and Rfam (http://rfam.sanger.ac.uk/) databases. The clean sRNAs were aligned within 3 mismatches in miRBase 21.0 (http://www.mirbase.org/ftp.shtml), and these were known miRNAs. For the novel miRNAs, the clean reads were reattached to the genome of *P*. *capsici* using Bowtie, and the maximum mismatch number in the seed sequence was allowed to be 0. Additionally, 1 read could be reposted to up to 5 different locations, and all identified miRNAs had to be able to fold into a hairpin structure predicted using miReap and miRDeep. The differential expression of known and novel miRNAs was analyzed using the Log2 ratio and scatter plots at the different stages.

### 2.4 Target prediction, functional analysis, and validation

The target genes of the differential miRNAs were predicted using miRanda and RNAhybrid. Gene ontology (GO) annotation analysis and (Kyoto Encyclopedia of Gene and Genome) KEGG pathway analysis were performed on the differentially expressed miRNAs at different stages. The interaction between miRNA and its target was verified using the tobacco transient expression system. The plasmids used for transient expression in plants were pEarlyGate100 and pENTR. The 3′ UTR sequence of the target gene was connected to the carboxyl end of eGFP and was inserted into the Entry vector pENTR, and then the sequence on the shuttle vector was transferred to the expression vector pEarlyGate100 by LR reaction. The miRNA mimic (synthesized using Guangzhou Ruibo) and *PcAGO1* were co-expressed in tobacco leaves, and 36 h after injection, the fluorescence signal intensity was observed and quantified using confocal fluorescence microscope and ImageJ, respectively. Ten views of each treatment were observed or quantified, and the representative images were shown.

### 2.5 Screening of functional microRNAs

The RT-qPCR method was used to verify miRNA expression in different isolates [LT1534; *PcAGO1*, *PcDCL1*, and *PcDCL2* knockout mutants; an empty vector isolate (EV) of *P. capsici*], which our laboratory obtained. Total RNAs were extracted from the different isolates using the TRIzol reagent. By adding a poly (A) tail at the end of mature miRNA and using an Oligo (dT) universal tag reverse transcription primer, cDNA was synthesized (TIANGEN, miRcute Plus miRNA First-Strand cDNA Synthesis Kit).

The reference gene for sRNA quantification in *P. capsici* was screened. *5S rRNA, 40S rRNA, 60S rRNA*, *GADPH*, *NU6,* and *NL5* were selected as candidates. The relative expression levels of other genes were normalized by choosing any 1 of the 6 genes as a reference gene. Primers of the miRNAs were designed as follows: The upstream primer was mature miRNA (U needed to be replaced by T). The downstream primer was a universal primer that bound to poly A. The real-time PCR reaction was performed with the SYBR reaction mix (TIANGEN, miRcute miRNA qPCR Detection Kit). Real-time PCR data were analyzed using the 2^−△△Ct^ method (Livak and Schmittgen, 2001).

### 2.6 MicroRNA origination analysis, microRNA knockout, and phenotype analysis

The sequences of miRNA were posted back to the genome using Bowtie. This is because a genome back-attached position sequence can form a secondary structure, and the sequence will not be a functional gene. About 300 bp sequences on both sides of the reposting sequence were knocked out. The sgRNAs for CRISPR/Cas9-mediated gene knockout was designed on EuPaGDT (http://grna.ctegd.uga.edu, accessed on 20 June 2021) and then cloned into pYF515 plasmids following a previously described protocol (Fang, et al., 2017). The sequences 1 kb upstream and 1 kb downstream of the target gene were amplified and cloned into the pBluescript II KS^+^ donor vector, and the replacement gene *NPTII* was inserted between them. The sgRNA and primers used in this study are listed in Supplementary Tables S1, S2. Gene disruption methods were performed using a CRISPR/Cas9-mediated gene replacement strategy (Fang and Tyler, 2016; Fang, et al., 2017). PCR was then used to detect miRNA precursors replaced by *NPTII* in the precursor-deleted transformants. Phenotypic characterization of the miRNA precursor transformants was done per Cui et al. (2023).

### 2.7 Data statistics

All experiments were replicated 3 times, each producing similar results. The data were analyzed using the one-way analysis of variance method, followed by Fisher’s least significant difference (LSD) test. The significance of mean separation was determined based on *p* < 0.05.

## 3 Results

### 3.1 Characterization of small RNAs from different stages of *Phytophthora capsici*

**FIGURE 1:**
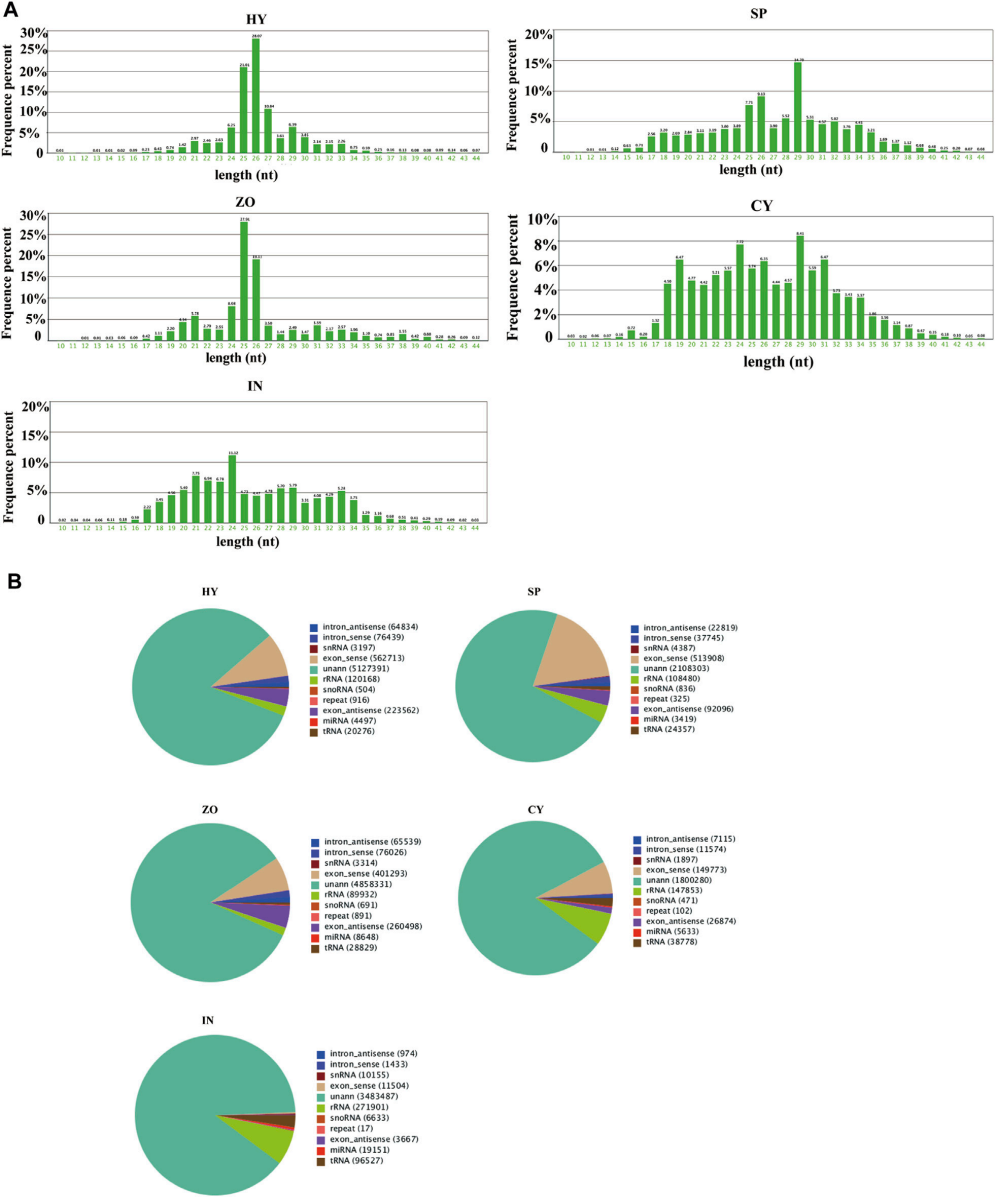
Characterization of small RNAs at different stages of *Phytophthora capsici*. **(A)** The length distribution of small RNA; **(B)** the mapping statistics of the unique reads. HY, mycelia; SP, sporangia; ZO, zoospore; CY, cyst; IN, pepper rhizome infected with LT1534 after 12 days.

To investigate the characterization of endogenous sRNAs from different stages of *P*. *capsici*, sRNA sequencing of all 4 asexual stages of *P*. *capsici,* with reference isolate LT1534, namely, mycelia (HY), sporangia (SP), zoospores (ZO), cysts (CY), and pepper rhizomes infected with LT1534 after 12 days (IN) (the severely diseased tissues contain sRNAs mainly derived from *P. capsici* but not plants, as mycelia were collected from diseased tissue. Besides, the sequences which only could be blasted to *P*. *capsici* genome but not *Capsicum annuum* genome were screened and further analysed as IN-stage sample.), was performed. The length distribution of sRNAs in these samples varied, as reflected by sRNAs being mainly distributed at 25–26 nt at the HY, SP, and ZO stages, but they were evenly distributed (18–34 nt) at the CY and IN stages. In addition, sRNAs at the SP and CY stages accumulated at 29 nt (Figure 1A). Classification of the sRNAs identified in the 5 samples was then performed. Common sRNAs existed in any pairs of stages, which showed that only 0.90%–10.92% of sRNAs could be identified in both samples. This indicated that most sRNAs are specifically expressed at different stages and that most sRNAs are unique at each stage (Supplementary Figure S1). Furthermore, more than 60% of sRNAs were unannotated, and among the annotated sRNAs, most originated from exon_sense or exon_antisense sequences and ribosomal RNA (rRNA) at all 5 stages. (These sRNAs were probably gene fragments but were not functional.) For canonical functional sRNAs, 0.072%, 0.117%, 0.149%, 0.257%, and 0.490% miRNAs (accounting for the total amount of sRNAs) were identified at the HY, SP, ZO, CY, and IN stages, respectively, which suggested that the proportion of miRNA in the total sRNAs was minute (Figure 1B). However, their biological roles in the development and pathogenesis of *Phytophthora* require further study.

### 3.2 Characteristics of known and novel microRNAs identified at the 5 stages of *Phytophthora capsici*

To explore the miRNAs of *P*. *capsici*, the annotated sRNAs were blasted with known miRNAs reported in the miRBase 21.0. The matched sRNAs were then designated as known miRNAs. Furthermore, for the sRNAs that could not be mapped to any miRNAs in the miRBase 21.0 but could be reattached to the antisense strand of the exons, introns, and intergenic region of the *P*. *capsici* genome, miRDeep and miReap were used to identify novel miRNAs using a strict hairpin structure analysis strategy. 92, 42, 176, 39, and 148 known miRNAs were identified in the 5 samples (HY, SP, ZO, CY, IN). Meanwhile, 15, 19, 54, 13, and 1 novel miRNA were identified at the HY, SP, ZO, CY, and IN stages, respectively. Pairwise comparisons between the 5 stages were performed to screen the differentially expressed miRNAs, of which 62 known miRNAs and 12 novel miRNAs with different expression levels were found in the SP compared to the CY group. Moreover, miRNA expression was more distinguished in the other groups of comparative samples, especially reflected by known miRNAs, which were all more than 100 (Table 1).

**TABLE 1:** The number of known and novel miRNA with differential expression in different stages of *Phytophthora capsici*.

Comparative samples	Known miRNA with significantly different expression level	Novel miRNA with significantly different expression level
HY vs. SP	109	17
HY vs. ZO	181	14
HY vs. CY	116	14
HY vs. IN	212	0
SP vs. ZO	183	26
SP vs. CY	62	12
SP vs. IN	175	0
ZO vs. CY	199	13
ZO vs. IN	299	0
CY vs. IN	176	0

HY, mycelia; SP, sporangia; ZO, zoospores; CY, cysts; IN, pepper rhizome infected by LT1534 after 12 days.

**FIGURE 2:**
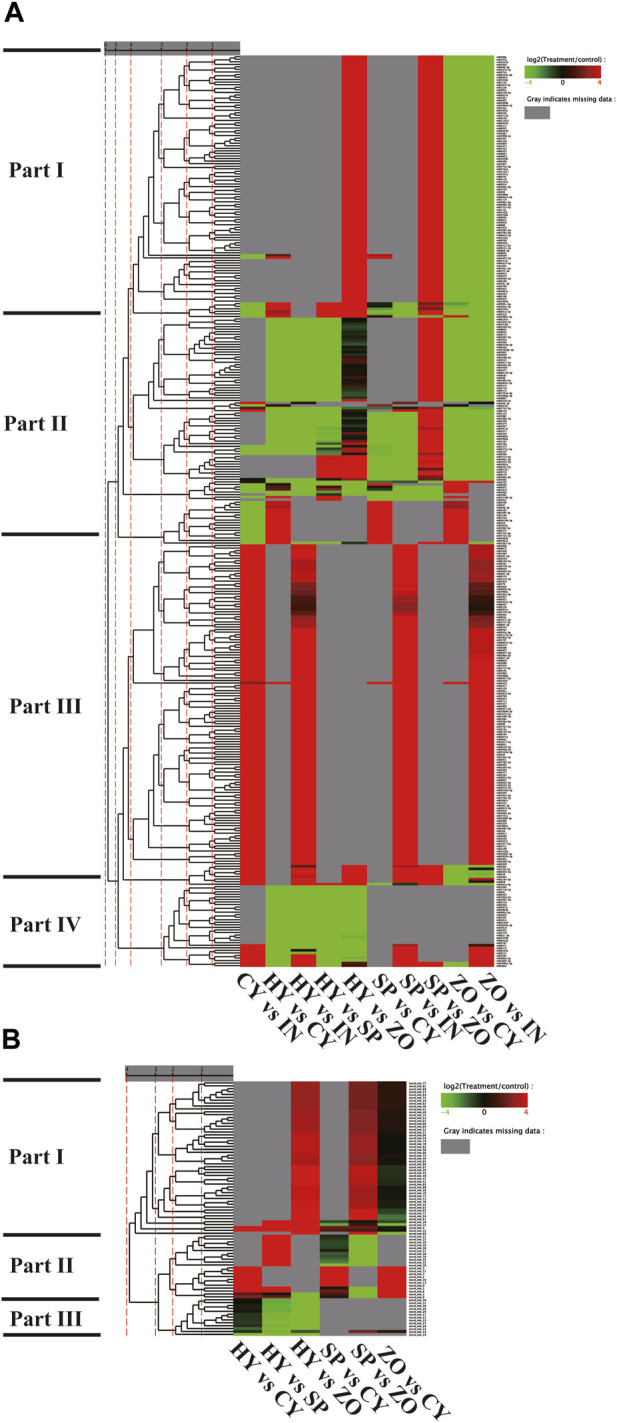
The expression pattern of known **(A)** and novel **(B)** microRNAs in any pairs of the 5 stages of *Phytophthora capsici*. HY, mycelia; SP, sporangia; ZO, zoospore; CY, cyst; IN, pepper rhizome infected with LT1534 after 12 days.

The detailed information of known and novel miRNAs differentially expressed in any pair of the 5 stages was analyzed. Since only 1 new miRNA was identified from the IN stage, no further analysis was performed on this stage. For the known miRNAs, the expression varied greatly at different stages, and they could be divided into 4 categories according to their expression pattern and miRNA family analysis. Part I (such as miR408a, miR1078, etc.), part III (such as miR399b, miR8672, etc.), and part IV (such as miR1040, miR171b-5p, etc.) were intensively expressed at the ZO, IN, and HY stages. Part II (such as miR1048-5p, miR1516d, etc.) was stably expressed at all stages but with different expression levels (Figure 2A). For novel miRNAs, the majority belonged to part I (such as novel-miR77, novel-miR81, etc.), which was highly expressed at the ZO stage and minimally expressed at the CY stage. Part II (such as novel-miR34, novel-miR8, etc.) was mainly identified at the CY and SP stages and was rarely detected at the HY and ZO stages. Part III (such as novel-miR18, novel-miR19, etc.) was only highly expressed at the HY stage (Figure 2B). These significant differences in the expression patterns of different miRNAs imply their role in *P. capsici* development and pathogenesis.

**FIGURE 3:**
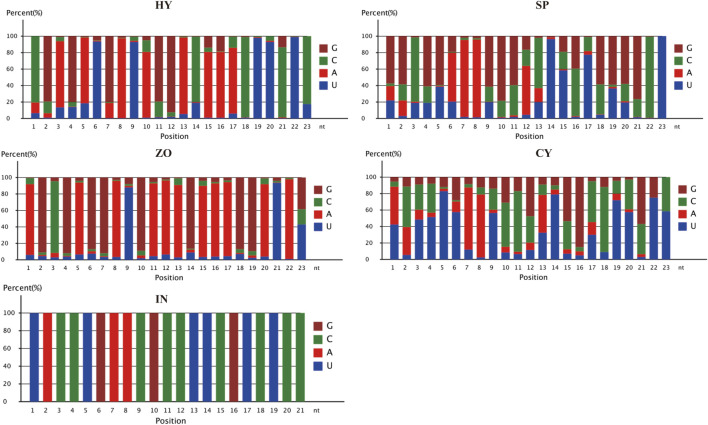
Nucleotide distribution at each position of the novel miRNAs at different stages of *Phytophthora capsici*. HY, mycelia; SP, sporangia; ZO, zoospore; CY, cyst; IN, pepper rhizome infected with LT1534 after 12 days.

The nucleotide distribution at each position of the novel miRNAs in HT, SP, ZO, CY, and IN was studied. C, G, A, and A was the most prominent 5′ nucleotide in HY, SP, ZO, and CY, respectively. An enrichment of A at the 8th position was observed at all stages, which suggested that A was the preferred nucleotide of endogenous miRNA at the 8th position in *P. capsici* (Figure 3).

### 3.3 Target prediction of microRNAs identified at various life stages of *Phytophthora capsici* and their gene ontology analysis

The biological function of miRNA is dependent on its regulation of target gene expression. Herein, we focused on the category and function of target genes potentially regulated by known and novel miRNAs, which were dynamically changed at the 5 stages. Nine to 52 species of known miRNA were detected at the 5 stages of *P. capsici*, and their potential target genes reached 13, 36, 81, 45, and 130 in HY, SP, ZO, CY, and IN, respectively. For novel miRNAs, 2, 5, 21, and 4 species corresponded to 8, 9, 39, and 7 target genes in the HY, SP, ZO, and CY samples (No novel miRNA was detected in the IN sample.) (Table 2).

**TABLE 2:** The number of predicted miRNA target genes in various stages of *Phytophthora capsici*.

Sample	Known miRNAs	Target genes of known miRNAs	Novel miRNAs	Target genes of novel miRNAs
HY	9	13	2	8
SP	34	81	21	39
ZO	52	130	0	0
CY	19	45	4	7
IN	14	36	5	9

HY, mycelia; SP, sporangia; ZO, zoospores; CY, cysts; IN, pepper rhizome infected by LT1534 after 12 days.

**FIGURE 4:**
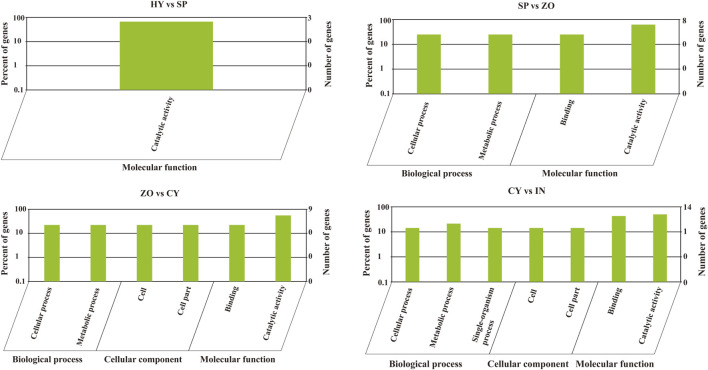
Gene oncology analysis of known microRNA-targeting genes at the different development stages of *Phytophthora capsici*. HY, mycelia; SP, sporangia; ZO, zoospore; CY, cyst; IN, pepper rhizome infected with LT1534 after 12 days.

*Phytophthora* undergoes a rapid and progressive lifecycle, which includes HY, SP, ZO, CY, and IN stages. These occur in a stepwise manner and are morphologically different. To explore the relationship between the development of different stages and miRNA-mediated gene regulation in *P. capsici*, we further analyzed the miRNA-regulated pathways that possibly participate in morphological transformations (HY to SP, SP to ZO, ZO to CY, and CY to IN). To begin with, the GO analysis of known miRNA-targeting genes showed that the catalytic activity pathway was the key to bridging mycelia growth and sporangia formation. During the latter transformations, the cellular and metabolic processes belonged to biological processes, and the binding function and catalytic activity belonged to molecular functions, which were continuously changed and possibly involved in these morphological transitions. In addition, during the transition from ZO to CY and from CY to IN, the cell and cell part-related components were also significantly changed, triggered by known miRNAs (Figure 4). Possibly because few target genes can be identified with novel miRNAs, the genes involved in certain GO pathways are rarely detected. Further, using KEGG analysis, we found that the targets of differentially expressed novel miRNAs were mainly concentrated in the lysine degradation pathway during the transition from HY to SP and from SP to ZO. Specifically, histidine to lysine N-methyltransferase was the key point regulated by novel miRNAs, which ultimately affected protein methylation and finally participated in the intricate morphological transition (Supplementary Figure S2).

### 3.4 Target gene expression validation and screening of functional microRNAs

To verify endogenous miRNAs in *P. capsici* and identify their biological functions, 8 known miRNAs that were highly and consistently expressed at different stages were selected for further analysis. For novel miRNAs, 8 species with at least 1 target gene were selected. The expression of the above candidate miRNAs was validated using quantitative reverse transcription–polymerase chain reaction (qRT–PCR), and their sequences are listed in Supplementary Table S3. Their expression levels in mixed samples of the HY, SP, and IN stages with LT1534, a *PcAGO1* knockout mutant (Ko*PcAGO1*), a *PcDCL1* knockout mutant (Ko*PcDCL1*), a *PcDCL2* knockout mutant (Ko*PcDCL2*), or EV were detected.

To begin with, the reference gene for sRNA quantification in *P. capsici* was studied. Six genes, namely, *5S rRNA*, *40S rRNA*, *60S rRNA*, *GADPH*, *NU6*, and *NL5*, which are reported to quantify sRNA in other organisms, were selected as candidates. The relative expression levels of other genes were normalized by choosing any 1 of the 6 genes as a reference gene. It was found that when *5S rRNA* was set as the reference gene, the standard deviation and variation of other genes were relatively small, indicating that as known stably expressed genes, they are least varied when using *5S rRNA* as the reference. Therefore, it was the most suitable reference gene for sRNA quantification in *P. capsici* (Supplementary Table S4).

**FIGURE 5:**
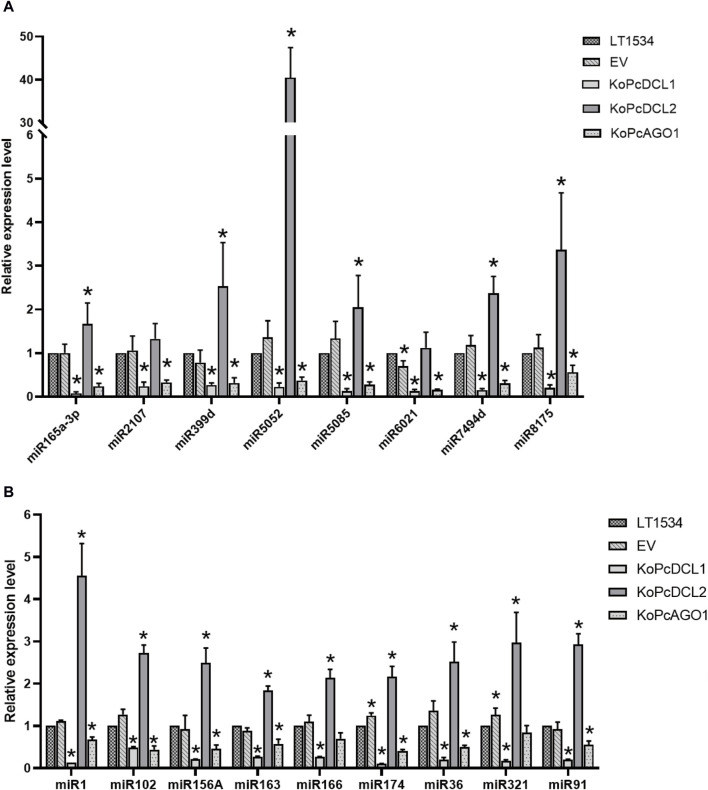
The relative expression level of candidate known **(A)** and novel **(B)** microRNAs in mixed samples of mycelia, sporangia, and the infection stage with LT1534, a *PcAGO1* knockout mutant, a *PcDCL1* knockout mutant, a *PcDCL2* knockout mutant, or an empty vector isolate, validated using quantitative reverse transcription–polymerase chain reaction.

As expected, most of the tested miRNAs were downregulated in the *PcDCL1* or *PcAGO1* mutants, which is consistent with the reports that *PcDCL1* and *PcAGO1* play important roles in miRNA processing. This result further indicated that the miRNAs that we screened were likely to be functional and true miRNAs. Most of the miRNAs were not downregulated in the *PcDCL2* mutant, indicating that *PcDCL2* is not directly responsible for the formation and function of miRNAs but may act on miRNA degradation or other sRNA-related pathways (Figure 5).

### 3.5 Generation and phenotypes of novel microRNA miR91 deletion mutants

MiR91 has more potential target genes than any other miRNAs, which imply that miR91 is extremely important. Here we chose miR91 as the representative to study the functional *Phytophthora* miRNA. The length of the mature miR91 was 24 nt, and the precursor of miR91 can form a canonical hairpin structure (Supplementary Figure S3), which was derived from PHYCAscaffold 164 and has only one copy in the genome. And its potential target genes, including 503142 (Inositol polyphosphate 5-phosphatase and related proteins), 505337, 505513, 529968, and 509270 (https://mycocosm.jgi.doe.gov/Phyca11/Phyca11.home.html), have been annotated as important genes. We next detected the expression level of 503142 and 509270 in *PcDCL1* knockout mutants, which demonstrated a significant upregulation of 503142 after the miRNA generation was disturbed (Supplementary Figure S4). These results further indicated that 503142 can be the target of miRNA(s). The precursor of miR91 was deleted using CRISPR/Cas9, and homozygous deletion mutants were obtained (Supplementary Figure S5). The mycelium growth, sporangia production, zoospore release, and cyst germination rates of wild-type LT1534, EV, and 5 deletion mutants were detected. The mycelial growth rate of the 5 mutants was significantly lower than that of the LT1534 and EV strains. 91-1-39, 91-2-1, and 91-2-5 also had lower sporangia production capacities than the LT1534 and EV strains, and the zoospore production of the 5 mutants was lower than that of the LT1534 and EV strains. The results thus showed that miR91 contributes to mycelial growth, sporangia production, and zoospore production in *P. capsici* (Table 3).

**TABLE 3:** Mycelium growth rate, sporangia production, zoospores releases, and cysts germination rate of wild type LT1534, EV, and five miR91 precursor knockout mutants.

Isolate	Mycelium growth rate (mm)	Sporangium production (per view)	Zoospore production (×10^4^/mL)	Cyst germination rate (100%)
WT	74.33 ± 1.90a	436.00 ± 47.13a	35.67 ± 11.71ab	0.77 ± 0.05a
EV	70.67 ± 1.27b	384.80 ± 94.13a	51.17 ± 17.66a	0.19 ± 0.05b
91-1-39	42.33 ± 1.48f	96.25 ± 57.52c	0.17 ± 0.37c	—
91-2-1	55.33 ± 0.87e	209.50 ± 78.90b	30.50 ± 8.50b	0.71 ± 0.01a
91-2-5	53.83 ± 1.24e	1.17 ± 1.46c	0.50 ± 0.76c	—
91-3-1	58.17 ± 1.73d	398.40 ± 91.93a	9.17 ± 4.37c	0.81 ± 0.16a
91-3-3	63.00 ± 1.07c	372.80 ± 100.60a	27.83 ± 14.26b	0.74 ± 0.06a

Means in a column with the same letter indicate no significant different according to a Fisher’s significant difference (LSD) test at *p* = 0.05.

### 3.6 Interaction validation between miR91 and its potential target gene

**FIGURE 6:**
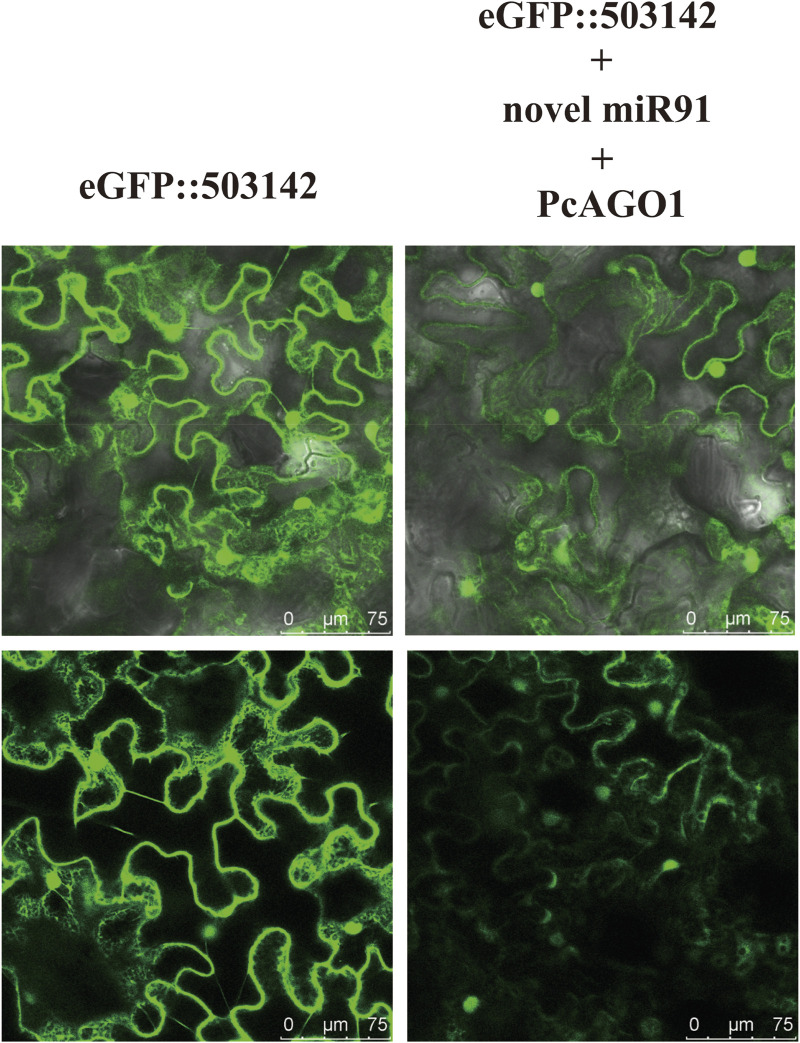
The interaction between miR91 and 503142, verified using the tobacco transient expression system. 503142 was predicted as a target of miR91, and its 3′ UTR was predicted to be bound by miR91. Expressing eGFP:3UTR in *Nicotiana benthamiana* (left), co-expressing eGFP:3 UTR and PcAGO1 in the presence of novel miR91 mimicked in *N*. *benthamiana* (right).

503142 was predicted as a target of miR91, and its 3′ UTR was predicted to be bound by miR91. The interaction between miR91 and 503142 was verified using the tobacco transient expression system. After the co-expression of miR91 and PcAGO1, the fluorescence intensity of 503142-3′UTR-tagged eGFP decreased, indicating that novel miR91 can repress the expression of 503142 by binding to its 3′-UTR. When the negative control mimic was added, the fluorescence intensity remained unchanged, which proved that the decrease in the fluorescence signal was specifically induced by miR91 and the fluorescence signal intensity was quantified using ImageJ (Supplementary Figure S6). Here, we verified the interaction between miR91 and its target gene (Figure 6).

## 4 Discussion

The asexual life stages are important for infection and disease development in *Phytophthora* species (Granke et al., 2012). Revealing the molecular mechanisms that regulate the lifecycle of *Phytophthora* is necessary for developing appropriate control strategies (Chen et al., 2013). However, there are few studies on the roles of endogenous sRNAs in the development or pathogenesis of *P. capsici*. In this study, it was found that sRNAs extracted from 4 asexual stages and infective stage were mainly distributed at 25–26 nt, which is significantly different from those of typical plant miRNAs but are similar to those of previous studies on *Phytophthora*. In *P. parasitica*, 25–26 nt sRNAs are mainly produced in gene-sparse and repeat-rich regions and are involved in regulating the expression of RXLR/CRN effectors and elicitor genes. These genes could not be detected at the HY and IN stages, indicating that 25–26 nt sRNAs are associated with the endogenous gene silencing of these genes (Jia et al., 2017). Consistently, studies have shown that 25–26 nt and 21 nt sRNAs are enriched in *P. infestans*, *P. sojae,* and *P. parasitica* (Vetukuri et al., 2012; Åsman et al., 2014; Jia et al., 2017). Furthermore, sRNAs were co-immunoprecipitated with 3 AGOs of *P. infestans* and then deep sequenced. The 25–26 nt sRNAs accumulated in AGO4, and 21 nt sRNAs accumulated in AGO1, demonstrating that these sRNAs are functional sRNAs (Åsman et al., 2016). In this study, these sRNAs with similar lengths also had abundance distribution, indicating that the sRNA in *Phytophthora* is evolutionarily conserved. Moreover, a high portion of 24 nt sRNA was detected, which is different from the known characteristics of *Phytophthora* sRNAs, may indicate that *P*. *capsici* sRNAs is different from other *Phytophthora* species. Two DCL proteins and a RdRP protein in *Phytophthora* are responsible for sRNAs generation, their expression pattern or sRNA sorting during different stages may be the key to result the sRNA distribution change.

Exploration of miRNAs is an efficient way to uncover the mystery of development, the function of miRNA in the development of model organisms is widely studied. For instance, *Drosophila* miRNAs positively regulate growth and the posterior signaling center (Lam et al., 2014), and dme-miR-14 regulates *Hedgehog,* which is involved in multiple developmental processes (Kim et al., 2014). During the development of the 4 larval stages of *Caenorhabditis*, 17 miRNAs regulate gene expression programs appropriate for different life history options (Karp et al., 2011). In our study, a large number of differentially expressed miRNAs were also identified at different stages of *P. capsici*. However, as there is only one known miRNA in *Phytophthora* and no predication standard could be used, many identified miRNAs may have been false positive predictions. In vertebrates, miRNAs have the following classic characteristics: They are produced from hairpin precursors by the Dicer endonuclease (Berninger et al., 2008), the secondary structure of hairpin precursors is stable, and there should be abundance in the sequencing data (Kang and Friedlander, 2015). At present, due to the lack of in-depth studies of the typical characteristics of miRNAs in microorganisms, the standard of miRNA identification is laxer than that of animals. However, a database could be constructed based on the sRNAs obtained in this study for explaining the relationship between the development of *Phytophthora* and sRNAs in the future. More accurate prediction standards for microbes should be created in future based on the identification of more “true” miRNAs, and the exact role of these miRNAs should be further explored.

In *P. infestans*, transcriptome of mycelia, sporangia, sporangia undergoing zoosporogenesis, motile zoospores, and germinated cysts were studied, respectively. It was found that the greatest changes occurred in the transition from hyphae to sporangia, where >4,200 genes were upregulated. Genes encoding calcium binding protein, the cation channel, signal protein, and flagellin were upregulated in sporangia. Genes related to pathogenicity were transcribed in a subclass-induced wave during zoosporogenesis, zoospores, or germinated cysts. Additionally, genes involved in most metabolic pathways were downregulated during cyst germination (Ah-Fong et al., 2017). GO analysis of the known miRNAs showed that they are mainly concentrated in binding and the catalytic activity pathway. There were a large number of cell component-related proteins regulated by differential miRNAs during the transition from ZO to CY and from CY to the IN stage, which may be related to the cell wall formation during the CY stage and plant cell wall degradation during the IN stage. This further indicates that sRNAs could be involved in the dramatic transcriptome changes during development and pathogenesis of *Phytophthora*.

Target gene is the necessary mediator for the function implement of miRNA, which could be bound by miRNA at its 3′ UTR region (Bandyopadhyay et al., 2015). Animal miRNAs target transcripts through imperfect base-pairing to multiple sites in 3′ UTR regions of a gene. However, plant miRNAs target transcripts with single and highly complementary target sites (Brodersen and Voinnet, 2009). Due to the lack of recognition and binding mechanism between microbial sRNAs and their targets, the target genes of miRNAs in microbes only could be predicted based on the experience and criteria obtained from animals and plants. In this study, the targets 503142 of the novel miRNA, miR91, were predicted and verified using the fluorescence reporting system. miR91 contributes to mycelial growth, sporangia production, zoospore production and virulence in *P. capsici*. Its target 503142 participates in intracellular trafficking, secretion, and vesicular transport as extracellular vesicles could transport small RNAs into pathogen cells to silence virulence-related genes (Cai et al., 2023). In summary, sRNAs play important roles in the development and pathogenicity of *Phytophthora*, they are involved in regulating of many genes in *P. capsici* spatio-temporally. The current work is benefit for the illumination of the development and infection mechanisms in *Phytophthora*.

## Data Availability

The data presented in the study are deposited in the SRA database [https://www.ncbi.nlm.nih.gov/bioproject/PRJNA1024393], accession number PRJNA1024393.
