# Supplementary material for: Distinct small RNAs are expressed at different stages of Phytophthora capsici and play important roles in development and pathogenesis
Source: Front Genet. 2024 Jun 11;15:1296533. doi: 10.3389/fgene.2024.1296533 (PMC11196614; doi:10.3389/fgene.2024.1296533)
Supplement: Supplementary file 1 [file Table1.DOCX]

Supplementary Material

# Supplementary Figures and Tables

## Supplementary Figures


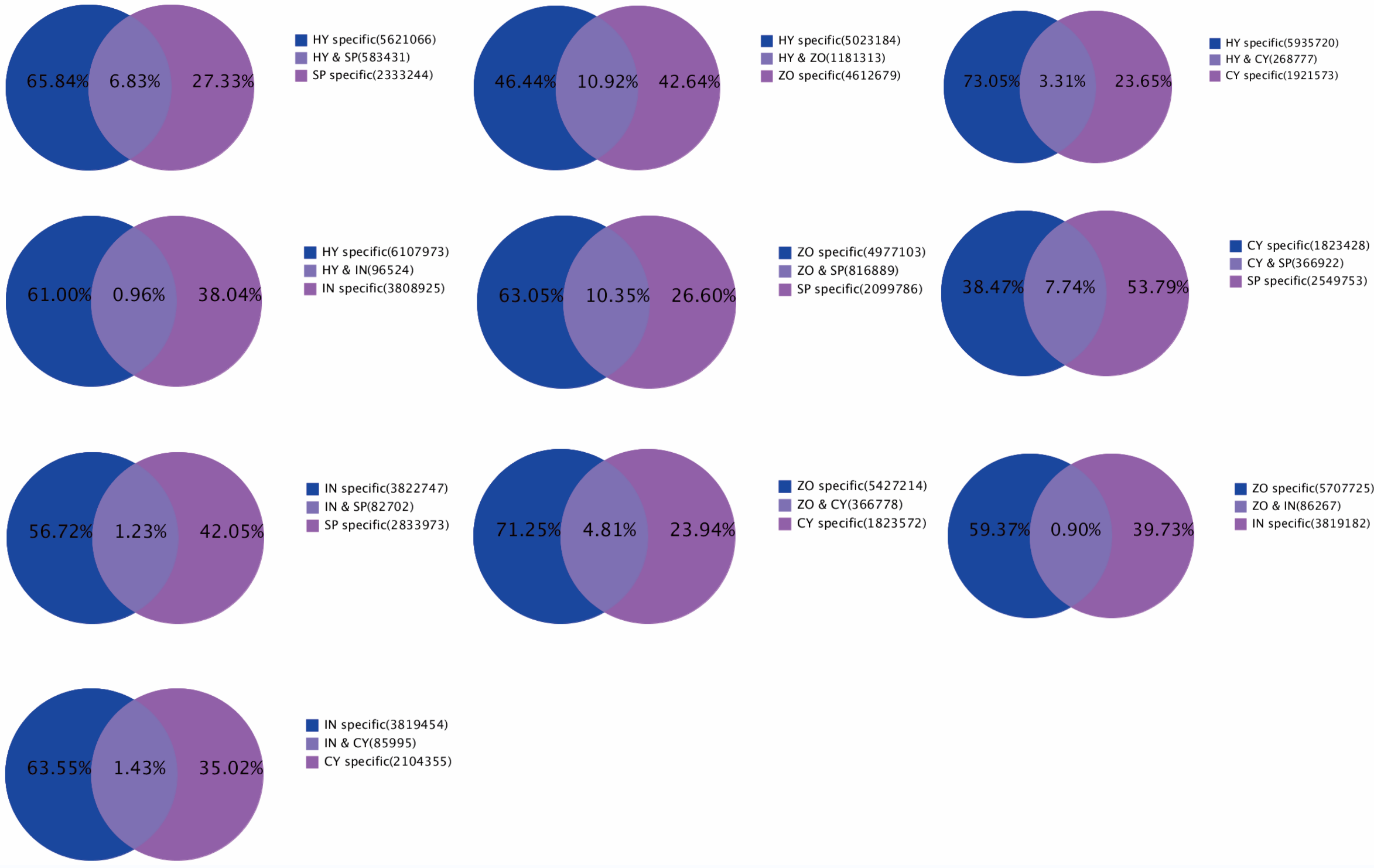


**Supplementary Figure 1.** Classification of sRNAs identified in different stages of *Phytophthora* *capsici*. HY: mycelia. SP: sporangia. ZO: zoospores. CY: cysts. IN: pepper rhizome infected by LT1534 after 12 days.


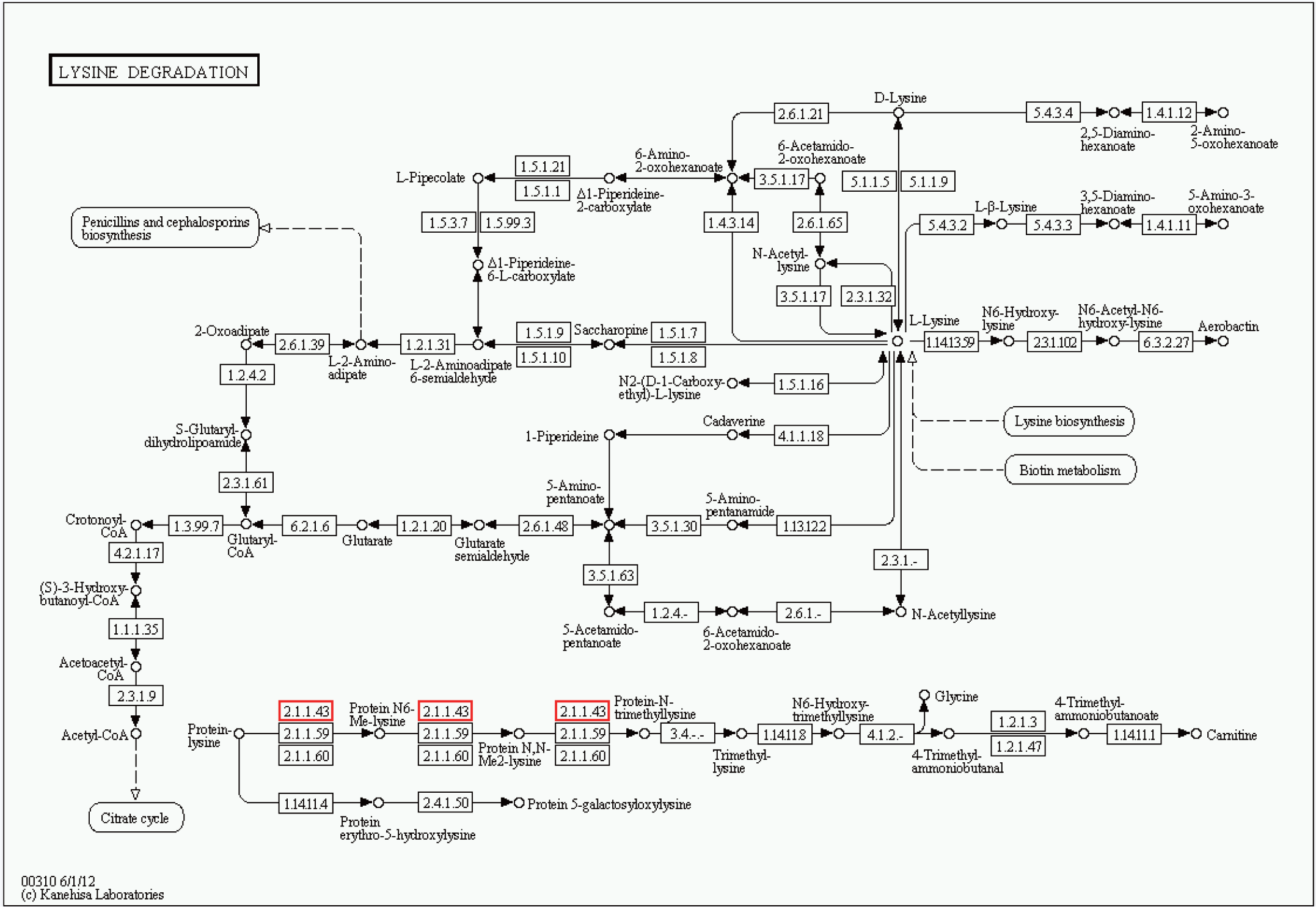


**Supplementary Figure 2.** KEGG analysis of novel miRNAs-targeting genes during the transition from mycelia to sporangia and from sporangia to zoospores stage.


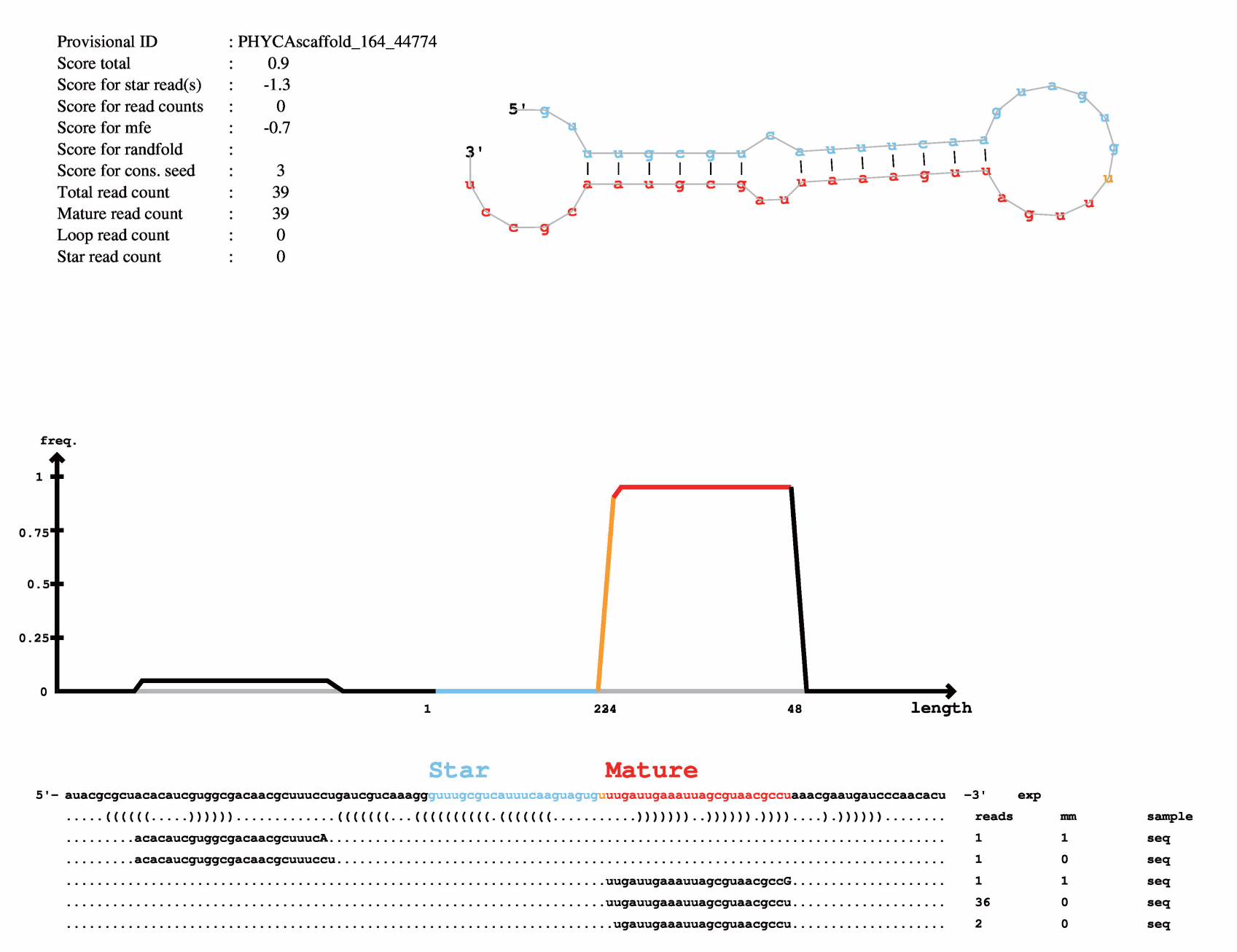


**Supplementary Figure 3.** A canonical hairpin structure of the miR91 precursor.


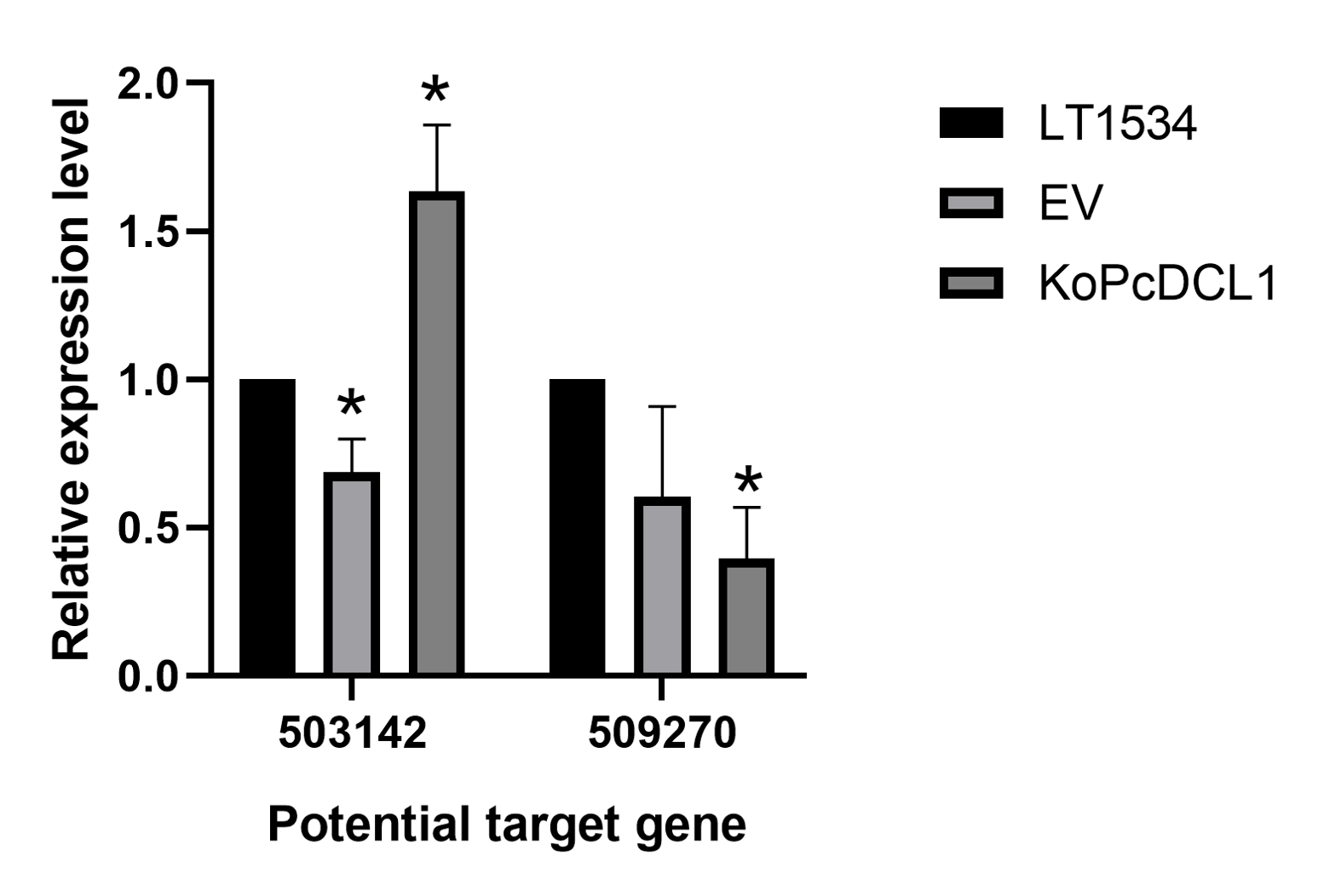


**Supplementary Figure 4.** The relative expression level of 503142 and 509270 in LT1534, empty vector isolate (EV) and *PcDCL1* knockout mutants (*KoPcDCL1*).


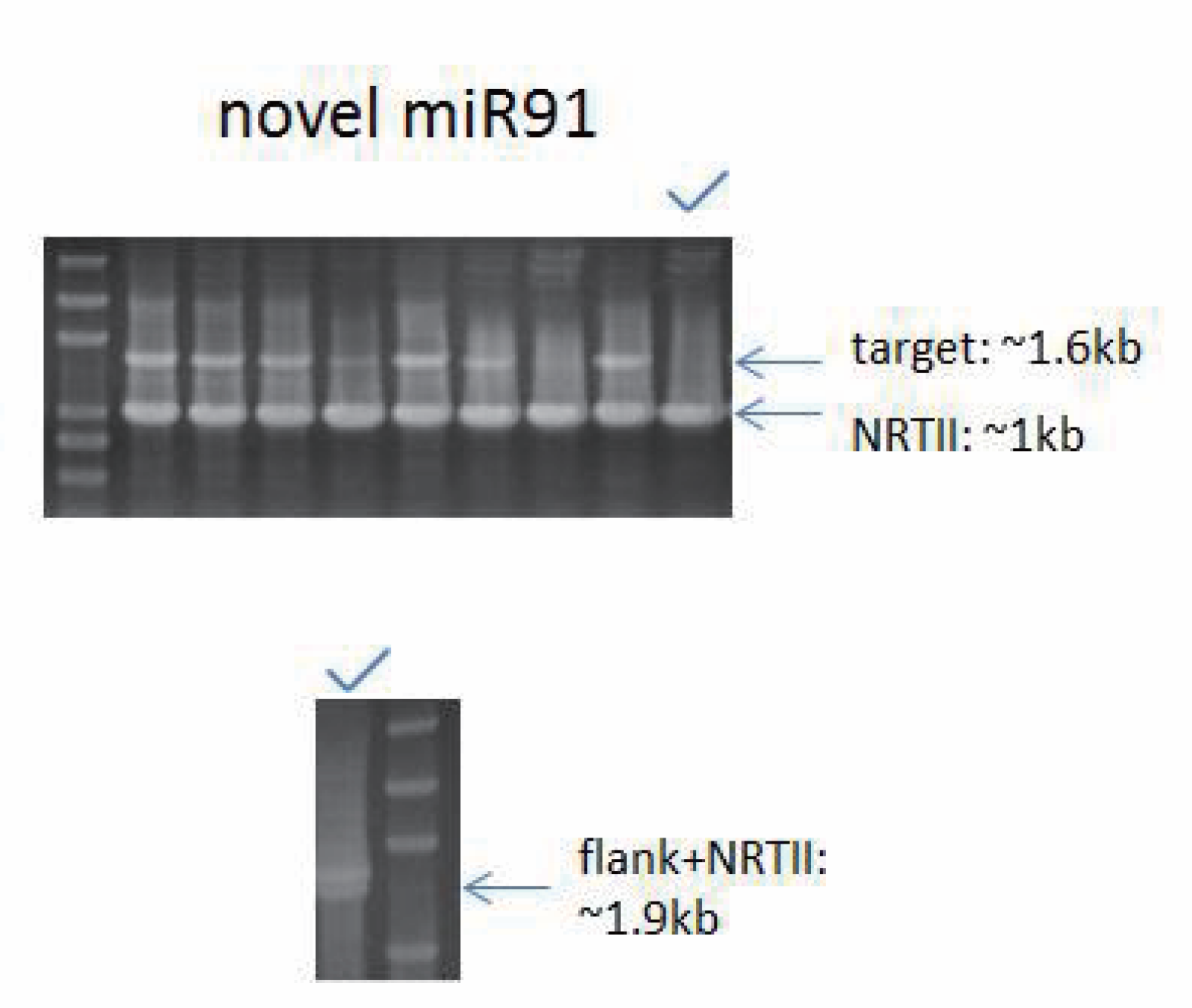


**Supplementary Figure 5.** The PCR verification of the miR91 precursor knockout mutants using CRISPR/Cas9.


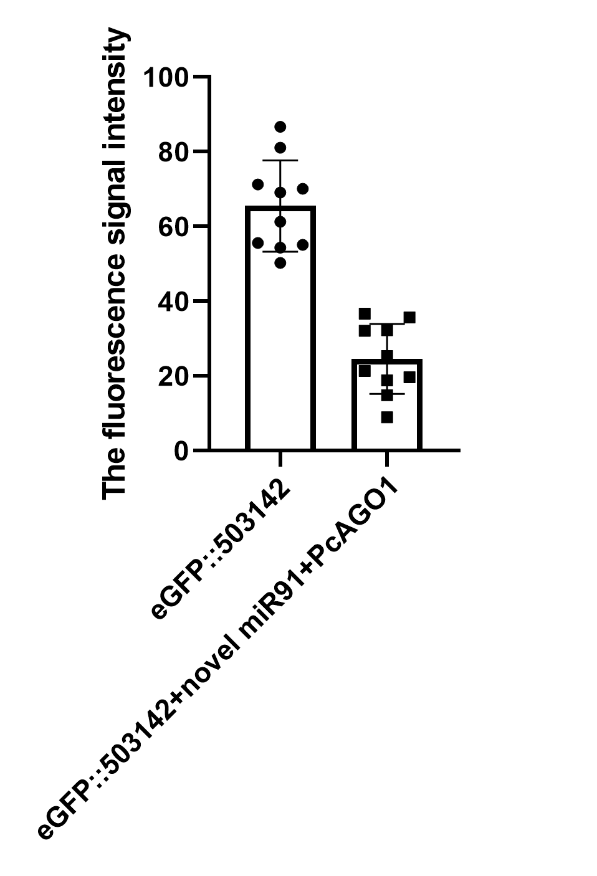


**Supplementary Figure 6.** The fluorescence signal intensity was quantified using ImageJ.

**
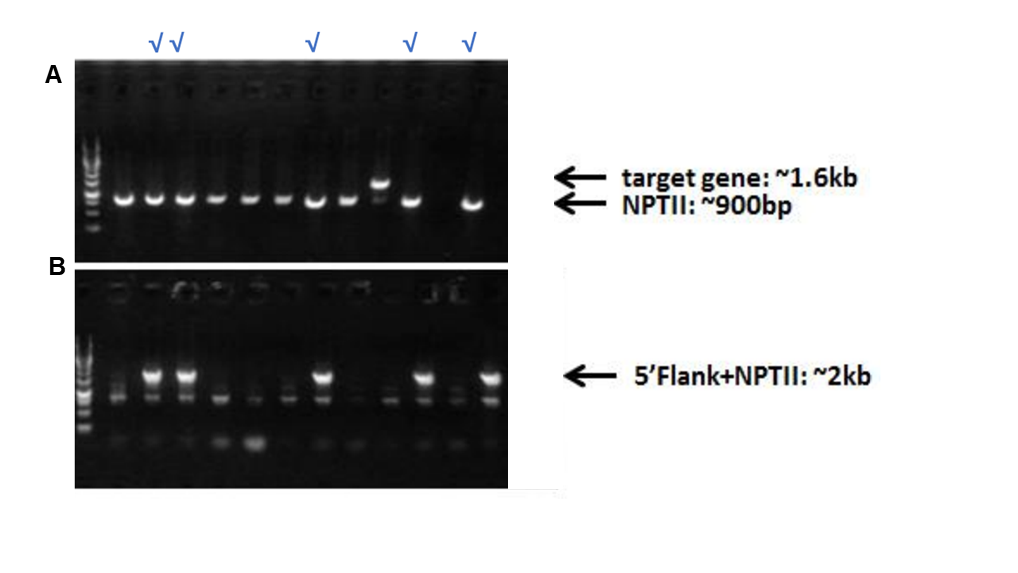
Supplementary Figure 7.** The PCR verification of the ΔPcAGO1, ΔPcDCL1 and ΔPcDCL2 mutants.

## Supplementary Tables

**Supplementary Table 1.** sgRNA sequence used in the study.

| Primer | Sequence (5’-3’) |
| --- | --- |
| 91-1F | CTAGCCTCTTCCTGATGAGTCCGTGAGGACGAAACGAGTAAGCTCGTCGAAGAGAATACCGTACCAGC |
| 91-1R | AAACGCTGGTACGGTATTCTCTTCGACGAGCTTACTCGTTTCGTCCTCACGGACTCATCAGGAAGAGG |
| 91-2F | CTAGCGTAGCCCTGATGAGTCCGTGAGGACGAAACGAGTAAGCTCGTCGGCTACTATCTCGTCGTTTC |
| 91-2R | AAACGAAACGACGAGATAGTAGCCGACGAGCTTACTCGTTTCGTCCTCACGGACTCATCAGGGCTACG |
| 91-3F | CTAGCTTTTACCTGATGAGTCCGTGAGGACGAAACGAGTAAGCTCGTCGTAAAATATGCGTCATAGAC |
| 91-3R | AAACGTCTATGACGCATATTTTACGACGAGCTTACTCGTTTCGTCCTCACGGACTCATCAGGTAAAAG |

**Supplementary Table 2.** Primers used in the study.

| Primer | Sequence (5’-3’) | Application |
| --- | --- | --- |
| U6F | AAGACACTGGTCGGTAAGGAGG | qRT-PCR analysis  of *NU6* |
| U6R | ATACTGGTCCACCGAATCCA |  |
| L5F | CGGTTTCGGTATCCAGGAGC | qRT-PCR analysis  of *NL5* |
| L5R | GCCGTAAATGCCCGTGGA |  |
| 40SF | CTGATCCGCAAGTGGCAGACTCTGATTG | qRT-PCR analysis  of *40S rRNA* |
| 40SR | GGCGTAGGTGGTCTTCTTGATCTGGTTC |  |
| 60SF | CCACCGTGACCGTATCTTCGCTATCCA | qRT-PCR analysis  of *60S rRNA* |
| 60SR | TCAGCGTAGTGAGCGGCAAGCATCT |  |
| 5.8S1F | CAGTAAGAATGGCTGGACGATGGA | qRT-PCR analysis  of *5S rRNA* |
| 5.8S1R | TTGCTCTCGATGTCTTGCTGTGT |  |
| GAPDHF | GCCAGCGAGAACGAGATGAAGG | qRT-PCR analysis  of *GADPH* |
| GAPDHR | AAGAGTGCGAGTCACCAATGAAGTC |  |
| 509270F | CCTGAACTACACTTCCACACAAG | qRT-PCR analysis  of 509270 |
| 509270R | CCTTCTTCAACAGCACAGC |  |
| 503142F | AAGAAGAACGAAGGCGAGA | qRT-PCR analysis  of 503142 |
| 503142R | ATACCCTGCTTCTGGTTCTGA |  |
| WS21F | GGAAAGAACAAACGCCTGAC | qRT-PCR analysis  of WS21 |
| WS21R | GTTGCGCTCCGAGAAGATA |  |
| 91-5F | CCCAAGCTTCCTGACTTCAGATTCTGTAGC | For miR91 donor  vector |
| 91-5R | CCGGAATTCCAATACTGACGAAAGACACCA | |
| 91-3F | GGACTAGTTCTGGGCTCTCCGTTG |  |
| 91-3R | ATAAGAATGCGGCCGCACACTTCCAGACGCTGC | |
| M13F | GTAAAACGACGGCCAGT |  |
| M13R | GGAAACAGCTATGACCATG |  |
| PV91F | CGCTCGTAGTGACTTCATTG | Verify the miR91 precursor knock out transformants |
| PV91R | AACCTTGTTGGACTCGTTTC |  |
| NPTF | TCTCCTGTCATCTCACCTTG |  |
| NPTR | ACCGTAAAGCACGAGGAA |  |
| 503142UF | CGGGATCCGCACTGCAGCGATAGTCGCT | For tobacco transient expression system |
| 503142UR | ACGCGTCGACTCGTAAACAGCTTAATCC |  |

**Supplementary Table 3.** The sequence of the candidate known and novel miRNAs.

|  | miRNA name | Sequence (5’-3’) |
| --- | --- | --- |
| known miRNA | miR165a-3p | TCGGACCAGGCTTCATCCCCC |
|  | miR2107 | CAACCTCTCGTGCCTGTACC |
|  | miR399d | TGCCAACGAGATGCCCCG |
|  | miR5052 | ACGCTGGCGGTAGGCCTA |
|  | miR5085 | AGGCATTTTTTGTGAGGCTG |
|  | miR6021 | TTGGAAGAGGACAGCATGGA |
|  | miR7494d | TCATGAGACTTTAGCTGCTG |
|  | miR8175 | GATCCCCGGACGGCGCACCA |
| novel miRNA | miR1 | TTCGAGAATTCTGTGGAAACTGATC |
|  | miR102 | CACTGAAATTCTGGCGAAAT |
|  | miR156A | TGACAGAAGAGAGTGAGCAC |
|  | miR163 | TACTTGAGTCCTCTGATTACTGCTT |
|  | miR166 | TCGGACCAGGCTTCATTCCCC |
|  | miR174 | TTGACGACGAGAGCTACAGGAAAT |
|  | miR36 | GAGGGGTCCACTGAAGAGCAC |
|  | miR321 | CGGTATTTGTCAGTGTAGGTCCAGCCC |
|  | miR91 | TTGATTGAAATTAGCGTAACGCCT |

**Supplementary Table 4.** The variation of reference gene for sRNAs quantification in *P*. *capsici*.

|  |  | Target gene | | | | | | Standard deviation | Mean value | Variation |
| --- | --- | --- | --- | --- | --- | --- | --- | --- | --- | --- |
|  |  | *5S rRNA* | *40S rRNA* | *60S rRNA* | *GADPH* | *NU6* | *NL5* |  |  |  |
| Reference gene | *5S rRNA* | - | 0.603 | 0.664 | 0.797 | 0.825 | 0.895 | 0.107 | 0.757 | 0.142 |
|  | *40S rRNA* | 0.650 | - | 0.218 | 0.399 | 0.492 | 1.203 | 0.336 | 0.593 | 0.567 |
|  | *60S rRNA* | 0.656 | 0.179 | - | 0.280 | 0.438 | 1.311 | 0.403 | 0.573 | 0.703 |
|  | *GADPH* | 0.927 | 0.350 | 0.333 | - | 0.386 | 1.159 | 0.345 | 0.631 | 0.547 |
|  | *NU6* | 0.827 | 0.798 | 0.769 | 0.577 | - | 0.974 | 0.127 | 0.789 | 0.161 |
|  | *NL5* | 1.114 | 1.124 | 1.044 | 0.907 | 0.685 | - | 0.164 | 0.975 | 0.169 |
| Standard |  | 0.175 | 0.333 | 0.299 | 0.235 | 0.165 | 0.153 | - | - | - |
| deviation |  |  |  |  |  |  |  |  |  |  |
| Mean value |  | 0.835 | 0.611 | 0.606 | 0.592 | 0.565 | 1.109 | - | - | - |
| Variation |  | 0.209 | 0.545 | 0.493 | 0.397 | 0.292 | 0.138 | - | - | - |
